# O-03 - A STRATEGY TO VISUALISE INFECTIOUS DISEASE NOTIFICATION DATA IN DYNAMIC CUSTOMISABLE DASHBOARDS FOR POLICYMAKERS, HEALTH PRACTITIONERS, ACADEMICS, THE MEDIA AND THE PUBLIC IN IRELAND

**DOI:** 10.1093/pubmed/fdag046.003

**Published:** 2026-07-09

**Authors:** Dr Patricia Garvey, Mr Eoghan McCarthy, Mr Cesar Alves, Anthony Ortiz, Kirsty MacKenzie, Kate Timoney, Ajay Oza, Emma O'Connor, Hub Working Groups

**Affiliations:** HSE Health Protection Surveillance Centre; HSE Health Protection Surveillance Centre; HSE Health Protection Surveillance Centre; HSE Health Protection Surveillance Centre; HSE Health Protection Surveillance Centre; HSE Health Protection Surveillance Centre; HSE Health Protection Surveillance Centre; HSE Health Protection Surveillance Centre; HSE Health Protection Surveillance Centre

## Abstract

**Background:**

The COVID-19 pandemic highlighted the importance of robust, accessible health statistics and their impact on real life health outcomes. Large volumes of infectious diseases notification data on cases and outbreaks are collected in Ireland under the Infectious Disease legislation. This information is used to enable immediate public health action, and to analyse trends and inform policy. Historically, Health Protection Surveillance Centre (HPSC) published these data in static pdf reports.

**Objectives:**

Since 2022, HPSC has focussed on a strategy to visualise notifiable disease data for stakeholders (policymakers, health practitioners, academics, the media and the public) using public-facing ARCGIS dashboards.

**Methods:**

For each hub, a multidisciplinary working group was formed, and hub design was informed by their knowledge of stakeholder requirements. HPSC created a dynamic set of visualisations for the available data using reproducible workflows to ensure consistent and timely dissemination to stakeholders.

**Results:**

Currently, on a weekly basis, case-based notification data are presented on the HPSC Weekly Notifiable Disease Hub. and on the HPSC Respiratory Virus Notification Hub. The Notified Outbreaks hub enables monitoring of trends in notified outbreaks. The hubs include features such as statistical measures to compare the current week's data with earlier comparable data, times trend representation, and filtering capacity to display data by disease group (for example, respiratory diseases, vaccine preventable diseases) and regional health area. Aggregate data from the hubs are available to download in .csv format.

**Conclusions:**

While there were challenges and compromises in displaying the required volume of data, we have succeeded in making available large volumes of infectious disease information from a single point of access in a dynamic customisable way to all our stakeholders, including citizen epidemiologists. The process also reflects our commitment to Open Data, as all data points presented on the hubs are available for download by uses directly from the hubs.

